# Clinical application of a contingent screening strategy for trisomies with cell-free DNA: a pilot study

**DOI:** 10.1186/s12884-019-2434-0

**Published:** 2019-08-01

**Authors:** María Ángeles Sánchez-Durán, Andrea Bernabeu García, Inés Calero, Jordi Ramis Fossas, Tamara Illescas, María Teresa Avilés, Nerea Maiz, Elena Carreras

**Affiliations:** 1 Maternal-Fetal Medicine Unit, Maternal-Fetal Medicine Department, Hospital Universitari Vall d’Hebron, Ps. Vall d’Hebron 119-129, 08035 Barcelona, Spain; 2 Universitat Autònoma de Barcelona, Barcelona, Spain; 3 Department of Laboratory, Hospital Universitari Vall d’Hebron, Barcelona, Spain

**Keywords:** Cell-free DNA, Contingent screening, Fetal trisomy, Prenatal screening

## Abstract

**Background:**

Different strategies have been designed for clinical implementation of cell-free DNA (cfDNA) testing. We aimed to evaluate the performance of a contingent strategy based on conventional screening and offering cfDNA to the intermediate-risk group, for the screening for trisomies 21, 18 and 13. Secondary objectives were to assess the uptake of cfDNA in women with intermediate-risk, to evaluate the performance of cfDNA testing, and the preferences of pregnant women with intermediate risk.

**Methods:**

Prospective observational pilot study between February 2016 and March 2017. Singleton pregnancies with a known outcome were included in the study. At the conventional screening (first trimester combined test or second trimester quadruple test) women were classified in high (risk ≥1:250) or low risk (< 1:250). For the study, a contingent strategy was applied: following the conventional screening women were classified into three groups: high risk (risk ≥1:10 or nuchal translucency ≥3 mm), intermediate-risk (risk 1:11 to 1:1500) and low risk (< 1:1500), and a cfDNA test was offered to those at the intermediate risk.

**Results:**

For the analysis, 2639 women were included, 2422 (91.8%) had a first trimester combined test and 217 (8.2%) a second trimester quadruple test. There were 5 cases of trisomy 21, 4 of trisomy 18 and none of trisomy 13. For the contingent strategy, the detection rate and false positive rates were 88.9% (8/9) and 1.3% (35/2630), respectively. For the conventional strategy, the detection rate and false positive rates were 66.7% (6/9) and 5.3% (140/2630), respectively. The cfDNA test had a detection rate for trisomy 21 of 100% (3 out of 3), and a false positive rate of 0.2% (1/466). In a survey, 81.8% (374/457) of women in the intermediate-risk group would choose cfDNA testing as the second line test, mainly due to the lack of risk for the fetus.

**Conclusion:**

A contingent screening strategy for trisomies 21, 18 and 13, based on conventional screening, and offering a cfDNA test to women with a risk between 1:11 to 1:1500, reduced the false positive rate and increased the detection rate for these trisomies. Moreover, this strategy is well accepted by women.

**Electronic supplementary material:**

The online version of this article (10.1186/s12884-019-2434-0) contains supplementary material, which is available to authorized users.

## Background

First trimester combined testing has become the standard screening strategy for Down syndrome, with a detection rate of 90% for trisomy 21, 95% for trisomies 18 and 13, and a false positive rate of 5% [1]. More recently, the study of cell-free DNA (cfDNA) in maternal blood has shown higher detection rates [2, 3], with an important reduction in false positive rates, minimizing the number of invasive procedures and their complications. The universal use of cfDNA testing is controversial due to concerns about cost. An alternative is a contingent strategy in two steps; firstly a conventional screening is performed, and secondly, a cfDNA test is offered only to those patients with intermediate-risk. This strategy would increase the detection rate and reduce the false positive rate [4].

The aim of the study was to assess the performance of the contingent screening for trisomies 21, 18 and 13. Secondary objectives were to assess the uptake of cfDNA in women with intermediate-risk, to evaluate the performance of cfDNA testing, and the preferences of pregnant women with intermediate risk.

## Methods

### Study design and population

This was a prospective observational study performed between February 2016 and March 2017 at Vall d’Hebron University Hospital, Barcelona, Spain. This was a pilot study, previous to the implementation of a contingent strategy based on conventional screening (first trimester combined test and second-trimester quadruple test) and offering cfDNA to the intermediate-risk group at the public health system in our region,

The study population was women referred consecutively to the hospital for aneuploidies screening. Eligible patients were at least 18 years of age, with a singleton pregnancy. Exclusion criteria were vanishing twin pregnancy and unknown karyotype or unknown neonatal phenotype. This study was approved by the institutional research ethics committee (CEIC-VHIR) and informed consent was obtained from all women included in this study.

#### Clinical protocol

As required in the regional prenatal screening protocol, all pregnant women were offered to screen for fetal trisomies at the first prenatal care visit. The first-trimester screening protocol included measurement of serum concentration of β-human chorionic gonadotropin (β-hCG) and pregnancy-associated plasma protein-A (PAPP-A) at 8 to 12 weeks, and an ultrasound scan at 11^+ 0^ to 13^+ 6^ weeks, where fetal crown-rump length [CRL] and nuchal translucency were measured. Subsequently, the risk for trisomies 21, 18 and 13 was calculated combining all these data, according to prenatal diagnosis protocol for fetal congenital anomalies [5]. A second trimester quadruple test, including measurement of serum β-hCG, alpha-fetoprotein, unconjugated-oestriol, and inhibin-A, was offered to those women attending the screening antenatal clinic after 13 + 6 weeks.

#### Conventional screening

**Fig. 1:**
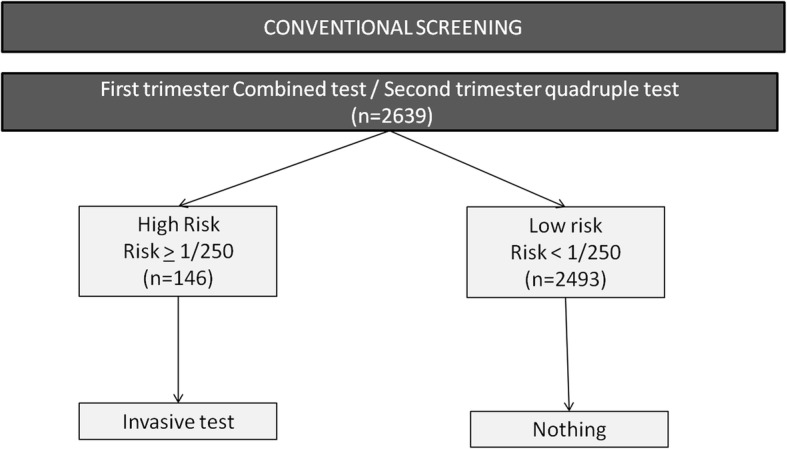
Conventional screening strategy

In the routine clinical practice, following first trimester combined test or second trimester quadruple test women were classified into two groups: high-risk, if the risk for trisomies 21, 18 or 13 was ≥1:250 or the nuchal translucency ≥3 mm, and low risk, if the risk was lower than 1:250. An invasive test was offered to women at high risk. In the low-risk group, no additional tests were offered and a second trimester scan was scheduled (Fig. 1).

#### Contingent screening (study)

**Fig. 2:**
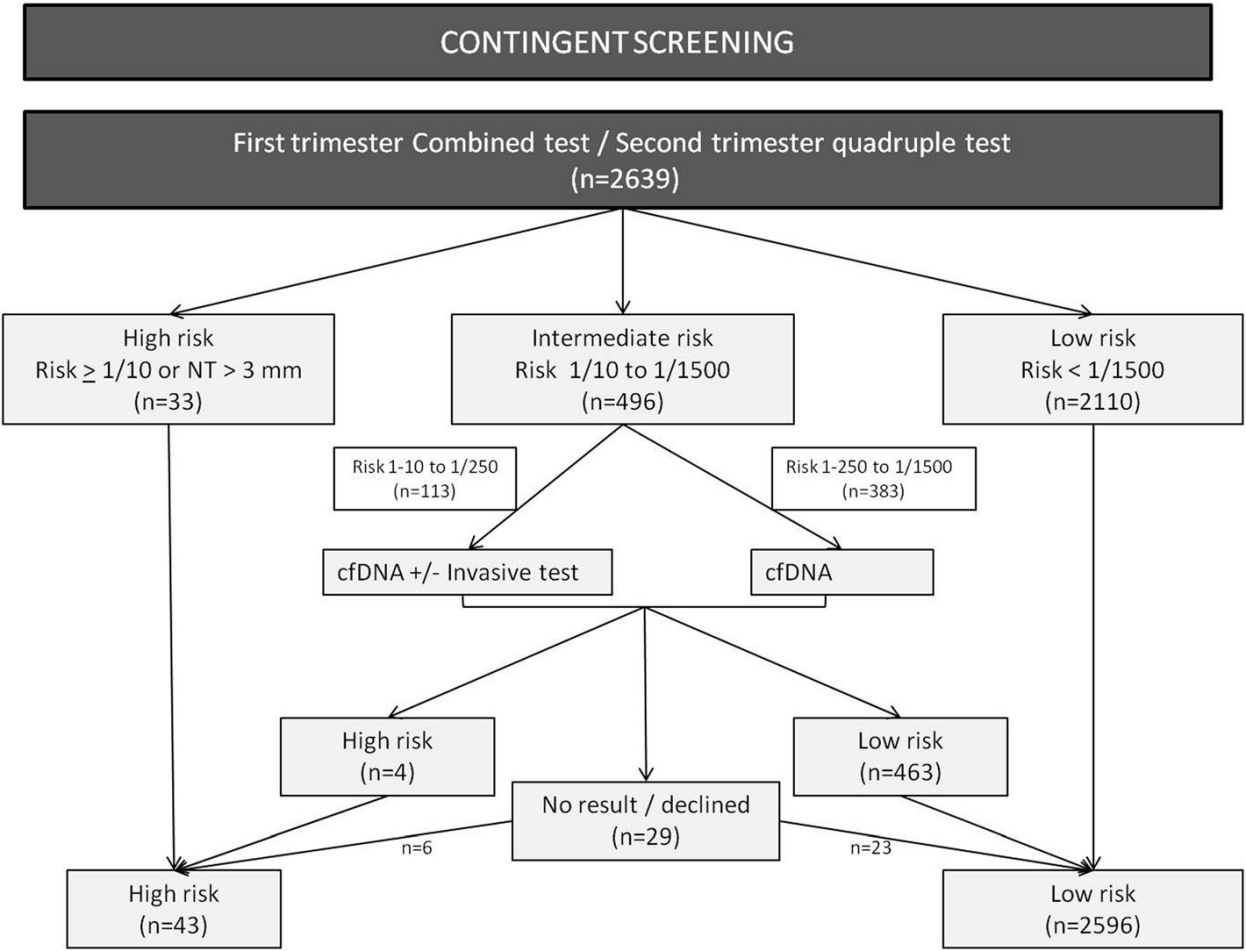
Contingent screening strategy

For the study, women were classified in three groups following the conventional screening: High-risk (risk ≥1:10 or nuchal translucency ≥3 mm), intermediate-risk (risk between 1:11 and 1:1500) and low-risk (risk < 1:1500). An invasive test was offered to women at high risk, no further test to women at low risk, and a cfDNA test was offered to those with intermediate-risk. Nevertheless, if the risk was between 1:11 and 1:250 additionally an invasive test was offered, according to the national screening protocol (Fig. 2).

A chorionic villus sampling or amniocentesis was carried out depending on gestational age. Prenatal diagnostic results of chromosomes 13, 18, 21, X and Y were released within 48 h of sample collection using QF-PCR and results of all chromosomal abnormalities were released with a time frame of 2–3 weeks by karyotype and/or array analysis.

Regarding cfDNA, peripheral maternal blood (20 mL) was collected into a standard ethylenediaminetetraacetic acid blood collection tube during the screening visit and sent via courier to the USA for cfDNA testing (Harmony® prenatal test, Ariosa Diagnostics, Inc., San Jose, CA, USA).

Genetic counseling was carried out by a trained obstetrician and screening results of the most common trisomies (21, 18 and 13) were expected to be obtained within 7–10 days. Women with a screen-positive result (high-risk result from cfDNA testing) were advised to have an invasive procedure in order to confirm the diagnosis, whereas women with a screen-negative result (low-risk result from cfDNA testing) continued conventional obstetric control during pregnancy as low-risk patients.

After cfDNA results delivery, women were asked to fill out an anonymous survey concerning the available contingent second-line options (Additional file 1).

All pregnant women were followed up until the end of pregnancy and all the newborns were studied for a phenotype by a pediatrician and a karyotype was requested if necessary.

### Study variables

Demographic maternal characteristics, including maternal age, weight, and race, were collected in the study electronic database by obstetricians during the screening visit. The risks for trisomies 21, 18 and 13 and the procedure performed after screening (invasive test, cfDNA or routine obstetric control) were collected in the database. We recorded the invasive test results, the cfDNA results (risk for trisomy 21, 18 or 13, fetal fraction in cfDNA from maternal plasma, test with no results, repetitions and time for result) and perinatal results of pregnancy.

The primary outcome of this study was the performance (detection rate and false positive rate) of the contingent strategy based on conventional screening and using cfDNA in the screening for trisomy 21, 18 and 13. Secondary outcomes were the detection rate and false positive rates of the cfDNA test, as well as the preferences of pregnant women.

### Statistical analysis

For the descriptive analysis, categorical data are shown as frequency and percentage, and continuous variables as the median and interquartile range (IQR). Pearson correlation was used to determine the association between fetal fraction and body mass index (BMI), and between the fetal fraction and the gestational age. Detection rate and false positive rate of both conventional and contingent screening strategies were calculated. Statistical significance level was set at *p* < 0.05. The statistical software package SPSS 23.0 (IBM SPSS Statistics for Windows, Version 23.0. Armonk, NY: IBM Corp) was used for the analysis of the data.

## Results

### Study population

**Fig. 3:**
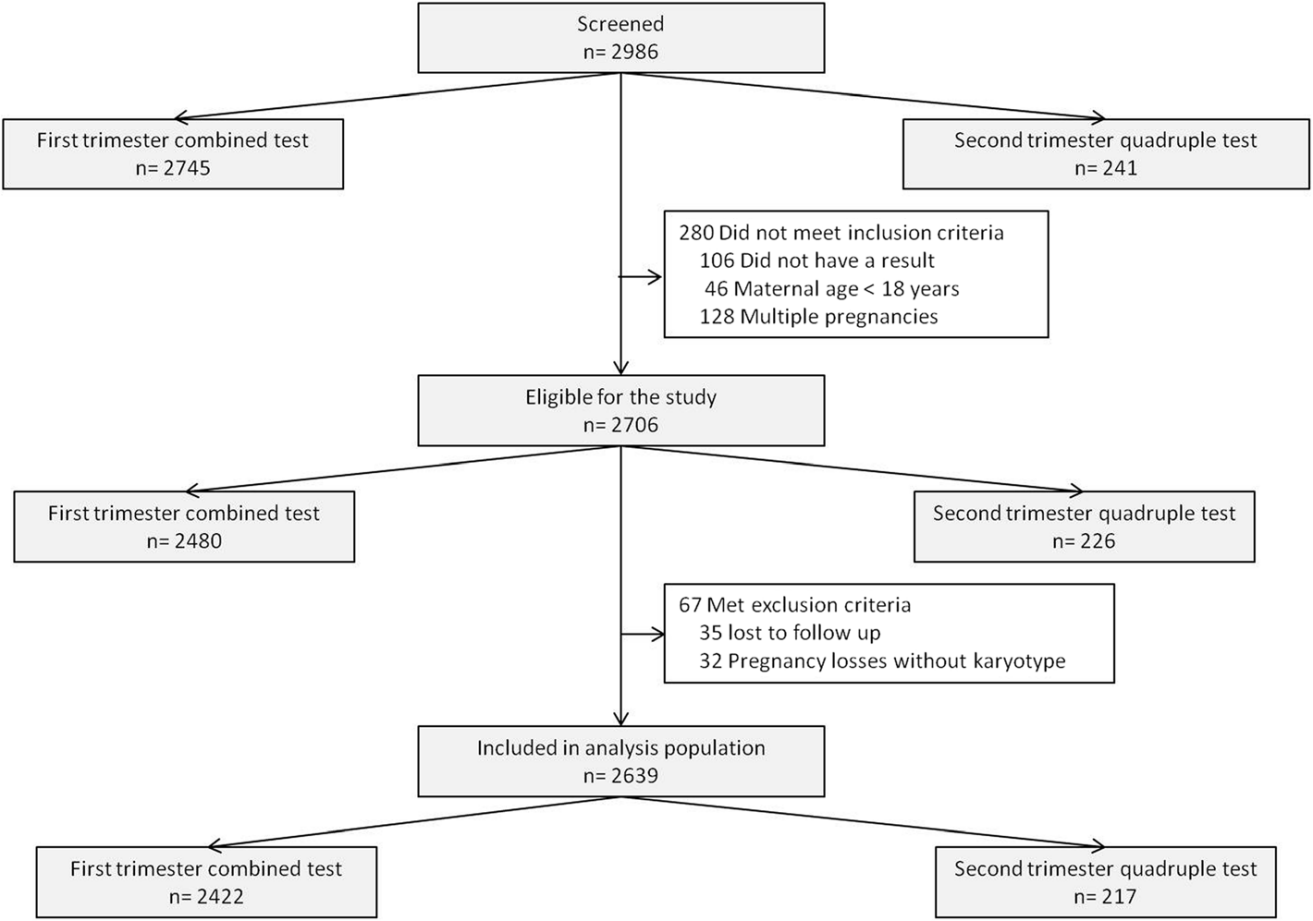
Enrollment

Two thousand nine hundred and eighty-six patients were screened in this period, 2706 women were eligible for the study, from these 67 (2.5%) were excluded, 2639 women were included for analysis, 2422 (91.8%) who had a first trimester combined test and 217 (8.2%) a second trimester quadruple test (Fig. 3). In this population, there were 5 cases of trisomy 21, 4 of trisomy 18 and no cases of trisomy 13 (Table 1).

**Table 1:** Description of the 9 cases of aneuploidies

Case	Aneuploidy	Screening Trimester	Risk 1 in	Conventional screening	cfDNA	Contingent screening
1	Trisomy 21	1	3	High risk	–	High risk
2	Trisomy 21	1	7	High risk	–	High risk
3	Trisomy 21	1	54	High risk	High-risk	High risk
4	Trisomy 21	1	596	Low risk	High-risk	High risk
5	Trisomy 21	2	741	Low risk	High-risk	High risk
6	Trisomy 18	1	1	High risk	–	High risk
7	Trisomy 18	1	1	High risk	–	High risk
8	Trisomy 18	1	1	High risk	–	High risk
9	Trisomy 18	1	13,735	Low risk	–	Low risk

*cfDNA* cell-free DNA

Median maternal age was 32.1 years (IQR, 28.1 to 36.0) and median weight 63 kg (IQR, 55.2 to 72.0). Two thousand three hundred and seven women (87.4%) were Caucasian, 131 (5.0%) North African, 73 (2.8%) Sub-Saharan, 71 (2.7%) East Asian, 51 (1.9%) South Asian and 6 (0.2%) mixed race.

### Conventional screening

According to the conventional test (first trimester combined test or second trimester quadruple test), 146 of 2639 (5.5%) women had a high risk (screen positive rate) and 2493 (94.5%) had a low risk (Fig. 1). The detection rate for all trisomies (trisomy 21, 18 and 13) was 66.7% (6/9) and the false positive rate 5.3% (140/2630). The specific detection rates for trisomy 21 and 18 were 60% (3/5), and 75% (3/4), respectively.

### Contingent screening

Following the conventional screening 33 (1.3%) women had a high risk (14 with a risk of 1:10 or more, and 19 fetuses with nuchal translucency of 3 mm or more), 496 (18.8%) patients had an intermediate risk (between 1:11 and 1:1500) and 2110 (79.9%) women had a low risk (< 1:1500).

From the 496 women with intermediate-risk 27 (5.4%) declined cfDNA testing, 7 had an invasive test (5 of them had a high risk), 2 had a cfDNA in a private center, one miscarried, one moved to another city, one refused due to difficulties for blood extraction and the other 15 refused to give a reason. In the 469 women who had a cfDNA test, 3 had a high risk for trisomy 21, one had a high risk for trisomy 13 and 463 had a low risk, and in two cases no result was obtained after repeating the test two and three times, respectively. The reason for the no result in both cases was a low fetal fraction. The risk for Down syndrome at the conventional screening in these two cases was 1:213 and 1:908, respectively. Both women who had no result and those who refused cfDNA testing were reclassified according to the result at the conventional screening. Following the cfDNA testing in the intermediate-risk group, 43 (1.6%) women had a high risk (screen positive rate), and 2596 (98.4%) had a low risk (Fig. 2).

In addition to the cfDNA test, an invasive test was offered to the group of 113 women with a risk between 1:11 and 1:250, 32 (28.3%) of them declined it and 81 (71.7%) underwent an invasive test.

For the contingent strategy, the detection rate for all three trisomies was 88.9% (8/9) and the false positive rate was 1.3% (35/2630). The specific detection rates for trisomy 21 and 18 were 100% (5/5) and 75% (3/4), respectively.

### Performance of cfDNA test

In the 469 women with intermediate-risk who accepted cfDNA testing, there were 3 cases of trisomy 21 and none of trisomy 18 or 13. In one case the result informed of high risk for trisomy 13, and the karyotype was normal. Median gestational age at cfDNA testing was 13.2 weeks (interquartile range, 12.5–14.1).

The detection rate for trisomy 21 was 100% (3 out of 3), and the overall false positive rate was 0.2% (1/466).

The test was repeated in 15 (3.2%) women, in 8 cases due to a sample mismanagement (4 cases where the samples were not labeled, 4 cases where the sample was lost during transport), 2 cases had a low fetal fraction and in 5 cases the samples did not meet quality criteria. In the second analysis, 12 cases showed a low risk for trisomies, and three samples again had no results, two of them were repeated, one had a low risk, and the other sample had no results for the third time. The latter was reported as a normal newborn at the end of follow-up. Regarding the sample which was not repeated, an invasive test was performed and the result was normal. The overall no-result rate was 0.43% (2/469). cfDNA results were issued after a mean of 6.7 (SD2.3) days, 90 and 87% of results were available within 8 and 7 days, respectively.

The median fetal fraction was 11.9% (IQR, 9.1 to 14.7). There was a significant correlation between fetal fraction and BMI, r  =  -0.364 (95% CI: − 0.356 to − 0.220; p  < 0 .001), and there was no significant correlation between fetal fraction and gestational age (r  =  0.086, 95% CI: − 0.013 to 0.354; p  = 0.068).

A total of 457 (97.4%) patients at intermediate-risk filled out the anonymous survey, the remaining patients of this group did not participate due to a language barrier. The results showed that 374 (81.8%) women would have preferred cfDNA testing as the second line contingent test, 80 (17.5%) would have preferred an invasive procedure, and 3 (0.7%) women not doing anything. The reasons for these answers are given in Table 2. Regarding the time for results, 239 women (52.3%) considered that it was short, 184 (40.3%) that it was appropriate, 30 (6.6%) thought that it was too long, and 4 (0.9%) did not respond to this question.

**Table 2:** Results of the survey

		Q1. Following the conventional screening, which procedure would you have preferred as the first option?		
		cfDNA (*n* = 374)	Invasive testing (*n* = 80)	No further testing (n = 3)
Q2.Why?	Diagnostic accuracy	16 (4.3%)	76 (95.0%)	0
	No risk for fetus	349 (93.3%)	1 (1.3%)	0
	Earlier diagnosis	2 (0.5%)	1 (1.3%)	0
	Other^a^	7 (1.9%)	2 (2.5%)	3 (100%)

*cfDNA* cell-free DNA

^a^ Other: cfDNA: 5/7 cases preferred to avoid a painful test; Invasive testing: did not give a specific reason; No further testing: 2/3 cases committed to the pregnancy. The rest of the cases did not specify a reason

Women’s opinion about available prenatal testing options

## Discussion

### Main findings of the study

This study confirms that a contingent screening strategy, based on the first trimester combined test or second trimester quadruple test and offering a cfDNA test to the intermediate-risk group, can improve the performance of the screening for trisomies 21, 18 and 13 by both increasing the detection rate and reducing the false positive rate. CfDNA was well accepted by this group of women, with an uptake of 94.6%, including women classified as ‘high risk’ by the conventional screening test. The main reason for accepting this test was the lack of risk for the fetus.

Focusing on the cfDNA test, the performance was excellent in this small sample, with a detection rate of 100% for trisomy 21, and an overall false positive rate of 0.2%. The no-result rate of the test was 0.43% after repeating the test up to three times. In a survey after the screening process, more than 80% of women in the intermediate-risk group would choose cfDNA testing as the first option, and more than 90% of women considered that the time to deliver the results was appropriate.

### Compare to the literature

Our findings are similar to those reported by other groups where a contingent screening, by means of a cfDNA test in the intermediate-risk group increases the detection rate and reduces the false positive rate [6, 7]. However, the performance and cost-effectiveness are difficult to compare among studies where different cut-offs have been used [8]. The cut-offs chosen in our study were 1:11 and 1:1500. When we have addressed the issue of implementing a contingent screening, we have set two goals: reducing the invasive test rate and increasing the detection rate. In order to reduce the invasive rate, we had to offer a cfDNA test to the high-risk group (≥1:250). We observed that most chromosomal abnormalities were in the group of risk of 1:10 or more, or in those with a nuchal translucency higher than 3 mm. As a consequence, we opted for this cut-off at the high-risk site. On the opposite side, we aimed to increase the detection rate and chose 1:1500 based on our own data and adjusting to our budget.

The no-result rate in our population was 3.2% following the first sample collection, and it was reduced to 0.43% after repeating the sample collection up to three times. Other studies have reported no-results rates for trisomies up to 5.9% [2].

It is important to highlight that the performance of the contingent strategy depends on the performance of the conventional screening (first trimester combined test or second trimester quadruple test), it is, therefore, important to maintain the standards of quality for these screening tests.

Previous studies have shown a reduction in the rate of invasive testing from 65 to 37% in the high-risk group (from the combined test) [6]. In our study, only 28% of women of this group opted to avoid invasive testing. We believe that this is explained by the lack of confidence of the women in the test due to the novelty.

### Strengths and limitations

The main limitation of this study is the sample size. The small number of affected fetuses in this cohort does not guarantee an accurate assessment and, therefore, a study with a larger sample size would ensure these results.

This study evaluated the feasibility of a contingent screening in real conditions, where 8% of women missed the first-trimester screening and had a second-trimester screening.

The uptake of our study is not comparable to other studies since this was a pilot study carried out under a regional clinical protocol, and women could not be deprived of the options included in this protocol, hence high-risk patients were offered an invasive procedure despite participating in the study and having a cfDNA test.

### Clinical implications

The confirmation of the good performance of the contingent strategy, the high uptake and good acceptance of the test by the patients will permit us to introduce the contingent screening in the clinical practice modifying the regional protocol. Invasive testing will remain as the first choice in cases of increased nuchal translucency, fetal structural abnormalities or risk higher than 1:10, and as a diagnostic test following a positive cfDNA result.

## Conclusions

A contingent screening strategy for trisomies 21, 18 and 13, based on conventional screening, and offering a cfDNA test to women with a risk between 1:11 to 1:1500, reduced the false positive rate and increased the detection rate for these trisomies. Moreover, this strategy was well accepted by women.

## Additional file

Additional file 1: Anonymous survey concerning available prenatal testing options. (DOCX 12 kb)

## Data Availability

The datasets used and analyzed during the current study are available from the corresponding author on reasonable request.

## Abbreviations

BMI: Body mass index
CfDNA: Cell-free DNA
CI: Confidence interval
CRL: Crown-rump length
IQR: Interquartile range
PAPP-A: Pregnancy-associated plasma protein-A
β-hCG: β-human chorionic gonadotropin
