# Supplementary material for: Clinical application of a contingent screening strategy for trisomies with cell-free DNA: a pilot study
Source: BMC Pregnancy Childbirth. 2019 Aug 1;19:274. doi: 10.1186/s12884-019-2434-0 (PMC6676567; doi:10.1186/s12884-019-2434-0)
Supplement: Supplementary file 1 — Anonymous survey concerning available prenatal testing options. (DOCX 12 kb) [file 12884_2019_2434_MOESM1_ESM.docx]

**Additional file 1**. Anonymous survey concerning available prenatal testing options.

1. *Following the conventional screening, which procedure would you have preferred as the first option?*
   1. Not doing anything
   2. cfDNA testing
   3. Invasive prenatal testing procedure
   4. I do not know risk and limitations of these procedures
2. *Why? Select only one option.*
   1. Diagnostic accuracy
   2. No risk for the fetus
   3. Earlier diagnosis
   4. Other (indicate cause):
3. *What do you think about the time for results?*
   1. Short
   2. Appropriate for diagnosis
   3. Too long
   4. Other

cfDNA: cell free DNA
